# Retraction: Long non-coding RNA PVT1-mediated miR-543/SERPINI1 axis plays a key role in the regulatory mechanism of ovarian cancer

**DOI:** 10.1042/BSR20200800_RET

**Published:** 2026-02-04

**Authors:** Chong Qu, Chunmei Dai, Yahua Guo, Rui Qin, Junbao Liu

**Keywords:** miR-543, ovarian cancer, PVT1, SERPINI1

This article is being retracted from *Bioscience Reports* at the request of the Editor-in-Chief and the Editorial Board. This follows the receipt of a notification from a reader, alerting the Editorial Board to concerns over the appearance of the apoptosis scatterplots of Figures 4E and 6F. Concerns include a ‘halo’ of regularly spaced dots and central areas with an unexpected texture. The Editorial Board note additional concerns over the article, including a lack of detail into which antibodies were used along with no mention in the author contribution of who performed the experiments. The authors have been contacted with regards to the retraction and have not responded to the Journal's queries or the concerns raised. Given the extent of the issues raised, the Editorial Board stands by the decision to retract the article.

